# The Impact of Hearing Aids on Listening Effort and Listening-Related Fatigue - Investigations in a Virtual Realistic Listening Environment

**DOI:** 10.1177/23312165241265199

**Published:** 2024-08-02

**Authors:** M. Blümer, J. Heeren, B. Mirkovic, M. Latzel, C. Gordon, D. Crowhen, M. Meis, K. Wagener, M. Schulte

**Affiliations:** 1 Department of Otorhinolaryngology, 37734 University Medical Center Hamburg-Eppendorf, Hamburg, Germany; 2 Hörzentrum Oldenburg gGmbH, Oldenburg, Germany; 3 Cluster of Excellence Hearing4All, Oldenburg, Germany; 4 Department of Psychology, 385626 University of Oldenburg School VI—Medicine and Health Sciences, Oldenburg, Germany; 5 87724 Sonova Holding AG, Stäfa, Switzerland; 6 Sonova New Zealand, Auckland, New Zealand

**Keywords:** listening effort, hearing aid use, ecological validity, listening-related fatigue, time compressed acoustic day (TCAD)

## Abstract

Participation in complex listening situations such as group conversations in noisy environments sets high demands on the auditory system and on cognitive processing. Reports of hearing-impaired people indicate that strenuous listening situations occurring throughout the day lead to feelings of fatigue at the end of the day. The aim of the present study was to develop a suitable test sequence to evoke and measure listening effort (LE) and listening-related fatigue (LRF), and, to evaluate the influence of hearing aid use on both dimensions in mild to moderately hearing-impaired participants. The chosen approach aims to reconstruct a representative acoustic day (Time Compressed Acoustic Day [TCAD]) by means of an eight-part hearing-test sequence with a total duration of approximately 2½ h. For this purpose, the hearing test sequence combined four different listening tasks with five different acoustic scenarios and was presented to the 20 test subjects using virtual acoustics in an open field measurement in aided and unaided conditions. Besides subjective ratings of LE and LRF, behavioral measures (response accuracy, reaction times), and an attention test (d2-R) were performed prior to and after the TCAD. Furthermore, stress hormones were evaluated by taking salivary samples. Subjective ratings of LRF increased throughout the test sequence. This effect was observed to be higher when testing unaided. In three of the eight listening tests, the aided condition led to significantly faster reaction times/response accuracies than in the unaided condition. In the d2-R test, an interaction in processing speed between time (pre- vs. post-TCAD) and provision (unaided vs. aided) was found suggesting an influence of hearing aid provision on LRF. A comparison of the averaged subjective ratings at the beginning and end of the TCAD shows a significant increase in LRF for both conditions. At the end of the TCAD, subjective fatigue was significantly lower when wearing hearing aids. The analysis of stress hormones did not reveal significant effects.

## Introduction

People with hearing loss have difficulty hearing and understanding speech in background noise (Dillon, 2012; Kramer et al., 2006). Besides deteriorated speech recognition, this group particularly shows increased listening effort (LE) compared to normal-hearing people (Krueger et al., 2017a; Schepker et al., 2016). The increased effort results from increased use of cognitive resources to complete a listening task, that is, the applied “mental effort” (Pichora-Fuller et al., 2016). Such mental effort arises when deteriorated listening-in-noise abilities are compensated for by, e.g., lip reading and top-down processing to ‘fill in the gaps’ caused by hearing loss (Hetu et al., 1988; Holman et al., 2019). In this context, among adults and children with hearing loss, sensations of tiredness, known as “fatigue” have been described (Bess & Hornsby, 2014; Hornsby et al., 2016; Key et al., 2017; McGarrigle et al., 2017). Fatigue includes the effects of chronic health conditions, which is then referred to as “long-term fatigue,” whereas “short-term fatigue,” or “transient fatigue” describes sensations of fatigue related to mental or physical demands and are typically resolved through rest (Hornsby et al., 2016). In this study, short-term fatigue that follows demanding listening tasks/listening situations was investigated, which is then referred to as listening-related fatigue (LRF). The link between LE and LRF was previously described and summarized by McGarrigle et al. (2014), Hornsby et al. (2016) and Pichora-Fuller et al. (2016). McGarrigle et al. (2014) describe “mental fatigue” as a consequence of effortful listening. Comparably, Hornsby et al. (2016) note that sustained, effortful listening can be fatiguing, and similarly, Pichora-Fuller et al. (2016) propose that fatigue develops because of the increased mental effort required for listening in challenging situations (i.e., LE), especially when the listener has hearing impairment. To approach the problem, the authors introduce a Framework for Understanding Effortful Listening, which integrates psychological theories of cognition and motivation to explain the effort required for listening. The framework integrates factors such as hearing difficulties, task demands, and the listener's motivation to expend mental effort in challenging situations. In essence, LRF is an accumulative effect of sustained LE over time, particularly when auditory conditions are adverse (e.g., difficult listening environments, impaired hearing).

The relevance of investigating fatigue and its individual and macroeconomic consequences is evident when considering its negative impacts in the areas of work performance, family life, and social relationships (Hornsby et al., 2016; McGarrigle et al., 2014; Pichora-Fuller et al., 2016). It is known that in comparison to their normal-hearing colleagues, individuals with hearing loss that experience LRF are more susceptible to workplace accidents, take more sick leave, and are more at risk to develop depressive symptoms (Hetu et al., 1988; Nachtegaal et al., 2009; Nachtegaal et al., 2012). The effect of interventions such as hearing aids (HA), cochlear implants (CI), or bone-anchored hearing aids (BAHA) on LE and LRF in patients’ daily lives is gaining increasing attention and was recently reviewed by Holman et al. (2021a). However, due to the heterogeneity of studies in this area, the authors were unable to perform statistically significant evaluations and emphasize that consistency is required when measuring LRF in future studies. The underlying methodological complexity, coupled with the absence of a common definition and standardized test procedures poses specific challenges for study design and data interpretation (McGarrigle et al., 2014).

Several studies assessed subjective levels of LE and LRF in the daily life of HA users (e.g., Alhanbali et al., 2017, 2018; Burke & Naylor, 2020; Davis et al., 2021; Holman et al., 2019, 2021b). A qualitative analysis by Davis et al. (2021) revealed the multidimensional nature of LRF in hearing-impaired people. According to their findings, the physical, mental, emotional, and social domain need to be taken into account when investigating LRF in daily life. The use of amplification was shown to have both positive and negative effects on LRF. Individuals reporting negative effects of HA usage on LRF reported they pick up much more sound than without their HAs. Burke and Naylor (2020) used ecological momentary assessment (EMA) to administer a questionnaire via a smartphone app by asking hearing-impaired and normal-hearing participants to report on their current listening situation and to provide a rating of their current level of fatigue at several quasi-random time points throughout the day. They found that through the day, both the hearing-impaired and normal-hearing participants became increasingly fatigued at a comparable rate. In a study by Alhanbali et al. (2017), asking for subjective ratings of self-perceived LE and LRF during daily life, the authors found that hearing-impaired participants reported significantly increased LE and LRF compared to the control group. Holman et al. (2019) focused on people with mild to moderate hearing loss, and conducted face-to-face, semistructured interviews. Though the link was weak, the majority of participants experienced lowered subjective LRF through HA usage. Another longitudinal study by Holman et al. (2021b) evaluated the effect of HA provision on LRF in hearing-impaired people by comparing an intervention- and a control group up to 6 months after they had received their first HAs. They found that LRF, but not general fatigue, was significantly lower in the intervention group compared to the control group. Additionally, social-activity levels increased and social participation restriction decreased significantly after HA provision. These examples from “real-world-measurement trials” highlight the potential of HA interventions on LRF. As a complement to measurement trials in the real-life environments of hearing-impaired people, studies in the laboratory have the potential to minimize deviations in LRF data that occur due to their multidimensional-nature (Davis et al., 2021). The controllability of the test environment in the laboratory enables cross-over study designs, where each participant experiences all test conditions with the same stimulation. However, the generalizability of laboratory tests to the everyday life of people is often limited. This aspect has been discussed extensively in the recent past (e.g., Beechey, 2022). In this context, the concept of ecological validity, which is defined as “the degree to which research findings reflect real-life hearing-related function, activity, or participation” (Keidser et al., 2020, p. 7S), aims at reducing differences between lab tests and real-life situations, using more realistic acoustical scenarios when testing.

The approach of this study was to reproduce a hearing-impaired person's “everyday-life listening” inside a laboratory test environment with the aim to assess the influence of HA usage on LE and LRF. Therefore, relevant listening situations were identified and implemented using virtual acoustics. Furthermore, suitable LE- and LRF-sensitive measurement methods were integrated into the test procedure. As the acoustical scene selection was based on daily-life scenarios, the whole test procedure was named Time Compressed Acoustic Day (TCAD, Heeren et al., 2020). In a survey conducted by Pang et al. (2019), the authors discovered that hearing-impaired participants reported speech understanding difficulties in: (1) busy environments, especially those with interfering speech, (2) situations with background music, (3) environments containing machine noise, (4) situations where the speaker is at a distance from the listener, (5) telephone signal quality, and (6) soft, unclear pronunciation. Jensen and Nielsen (2005) and Wagener et al. (2008) reported similar results. Schulte et al. (2013) and Appleton (2022) evaluated the importance of specific listening scenarios for HA users, and found that news broadcasts, as well as trying to understand speech from an adjacent room, are further important everyday life situations at home that can pose challenges for the hearing-impaired listener. In addition to impaired auditory abilities and the adversity of a listening situation, individual motivation to follow a conversation or, in the lab, to complete a listening task, plays an important role in the willingness to exert effort when following a conversation or a target speaker (Pichora-Fuller et al., 2016). As Hockey (2011) stated in “A motivational control theory of cognitive fatigue,” performance decrements can also result from boredom or low intrinsic attractiveness of the task itself, rather than from the difficulty of meeting its demands. That has given cause to vary listening situations and methods/tasks in the TCAD in order to provide novelty, thus aiming to keep the participant's motivation high during test sessions.

For LE, the literature suggests methods and tasks ranging from scaling procedures (Krueger et al., 2017a; Krueger et al., 2017b), pupillometry (e.g., Naylor et al., 2018; Neagu et al., 2023; Wendt et al., 2017; Winn et al., 2018; Zekveld et al., 2011, 2019), and electroencephalography (EEG; e.g., Mackersie & Calderon-Moultrie, 2016; Obleser & Weisz, 2012; Winneke et al., 2020) to reaction times (RTs) (e.g., Houben et al., 2013; Picou & Ricketts, 2014). Scaling procedures collect subjective judgments of LE for low and high signal-to-noise ratios (SNR). In dual-task paradigms, an increase in RTs is interpreted as a performance decline that results from an increased demand of cognitive resources (Houben et al., 2013; Picou & Ricketts, 2014). This is associated with an increase in LE. Feuerstein investigated the effect of a simulated hearing loss in a visual RT task (Feuerstein, 1992) and found that LE was significantly increased as indicated by increased RTs. Also, HA noise reduction algorithms were investigated and lower RTs were found when they were activated (Sarampalis et al., 2009). An overview of established methods for measuring LE and how to apply them can be found in a recent publication by Richter et al. (2023) “Combining Multiple Psychophysiological Measures of Listening Effort: Challenges and Recommendations.”

To assess the impact of HA usage on LE and LRF, Hornsby (2013) conducted a study with HA users having mild to severe sensorineural hearing loss, in unaided and aided test conditions. The study employed a 1 h, dual-task paradigm, assessing word recognition, memory recall, and RT, as well as subjective evaluations of attentiveness and fatigue. Additionally, the influence of advanced HA features, such as directional microphones and digital noise reduction, was tested. The test results showed significantly better word recall and faster RTs when testing aided. Subjective ratings of fatigue increased, and rated attentiveness decreased significantly after completion of the task, but no differences between unaided and aided testing were observed. Thus, while the behavioral results imply less need to invest LE when wearing HAs, and perhaps less LRF as a result, such effects were not confirmed in the subjective ratings. Furthermore, advanced signal-processing strategies did not show additional benefits in terms of reduced LE or LRF. The nonobserved benefit due to advanced signal processing strategies seems unexpected given the number of studies showing a decrease in LE when signal enhancement algorithms are activated (e.g., Alcántara et al., 2003; Brons et al., 2013, 2014; Luts et al., 2010).

The stress hormones cortisol and alpha-amylase have extensively been used as psychophysiological markers of stress in psychological studies (e.g., with the “Trier Social Stress Test”, Ali & Nater, 2020; Kirschbaum et al., 1993). Cortisol is a biomarker of the stress sensitive hypothalamic–pituitary–adrenal (HPA) axis whereas alpha-amylase is a biomarker of the autonomic nervous system (e.g., Engert et al., 2011; Zekveld et al., 2019) and as stress is often associated with fatigue, these noninvasive biomarkers were included. However, the relationship with LE and LRF is currently unclear (for a review, see Hornsby et al., 2016). Bess et al. (2016) measured the diurnal salivary cortisol profiles of groups of children on school days and found an increased cortisol awakening response in hearing-impaired children compared to children with normal hearing. This pattern suggests a potential dysregulation in the stress response system among children with hearing loss. Similar but nonsignificant results were found by Hicks and Tharpe (2002), suggesting that cortisol levels can be studied as a marker of long-term chronic fatigue. However, the sensitivity of this measure for detecting more transient fatigue, particularly task-related responses, remains unclear (Francis & Love, 2019; Hornsby et al., 2016). In a laboratory study, Zekveld et al. (2019) compared a listening condition with no feedback or social pressure to a condition with feedback and increased social pressure for improved listening performance. They expected that the stress manipulations would increase the motivation of the listener to perform the test successfully, and that this would be reflected in a larger pupil-dilation response and higher stress-hormone levels. Cortisol and alpha-amylase activity, as determined in saliva samples, did not, however, differ between the feedback and control groups. While previous laboratory studies on effortful listening, which examined stress hormones, had a maximum duration of around 1 h, the TCAD design comprises approximately 2½ h of measurement time. This could provide a new perspective on the investigation of stress hormones in effortful laboratory listening conditions.

To gain insights into the neuronal brain activity during effortful listening, EEG was recorded continuously during the whole test sequence. Therefore, one of the TCAD listening tasks was specifically designed to investigate auditory attention decoding (AAD). AAD relies on neural tracking of the stimulus and entails an analysis of neural responses to the envelope of continuous stimuli (Ding & Simon, 2012; O'Sullivan et al., 2015; Petersen et al., 2017). When speech is presented at low SNR, the AAD component decreases (Petersen et al., 2017) and its latencies increase (Mirkovic et al., 2019). Jaeger et al. (2020) found that when LE was reported to be high, the AAD components tended to be less prominent. Subjects with poor decoding performance also showed higher fatigue ratings.

The TCAD was comprised of a sequence of behavioral tasks. As RTs were previously shown to be sensitive to LRF, a dual task based on the study by Hornsby (2013), as well as an auditory comprehension task (single-task paradigm), were implemented. Furthermore, an informationally complex task with varying target talkers was included. Subjective ratings of LE and LRF were assessed after each listening task. To assess the overall effect on cognitive performance, a visual attention test was carried out at the start and the end of the TCAD.

### Hypotheses

As a wide variety of measurement methods were used, the corresponding hypotheses were formulated:

1. Subjective LRF ratings will increase throughout the TCAD.
2. Behavioral measures, such as RTs and word recognition, will decline during the TCAD.
3. LRF will lead to a general cognitive performance decrease, also in a visual attention test.
4. Stress hormone levels will increase with ongoing test duration.
5. Using HAs will lead to less LRF compared to unaided testing.

Another hypothesis was that LRF will lead to decreasing AAD components in the EEG analysis. This effect was suspected to be more pronounced when testing unaided. Due to technical problems resulting in data loss, only a part of the EEG data could be analyzed. The data can be found in the Appendix.

## Methods

### General Design and Procedure

Various listening situations were paired with LE and LRF measurement techniques. Altogether, five different acoustical scenarios were combined with four different listening-test paradigms. Each listening situation had a duration of approximately 15 min. Tests were repeated during the TCAD, and a complete session took 150–160 min. Table 1 provides an overview of the chronological arrangement of the sequence, including test name, acoustical scenario, test procedure, and test duration.

**Table 1.** Test Name, Acoustical Scenario, Description, and Duration of all TCAD Tests in the Order as Presented.

Test number: name	Acoustical scenario	Test procedure	Time (min)
T1: saliva sample	No background noise	Stress hormones (cortisol, alpha-amylase)	0–4
T2: d2-R	No background noise	Character scanning accuracy (visual)	5–14
T3: OLSBY I	Cafeteria background noise	Memory recall (auditory), response time (visual)	15–31
T4: OLERT I	Quiet home situation with soft background sounds	Response time to target words in running speech (auditory)	32–46
T5: saliva sample	No background noise	Stress hormones (cortisol, alpha-amylase)	47–51
T6: attended speaker I	Radio with traffic news background, speech from different room	Speech in speech, comprehension task (auditory)	52–64
T7: OLSBY II	Cafeteria background noise	Memory recall (auditory), response time (visual)	65–81
T8: CCOLSA	Public-house background noise	Word recognition of switching target talkers (auditory)	82–98
T9: saliva sample	No background noise	Stress hormones (cortisol, alpha-amylase)	89–103
T10: OLERT II	Living-room scene with radio traffic news from an adjacent kitchen	Response time to target words in running speech (auditory) → Directly streamed to the participant's HAs when performing aided	104–116
T11: OLSBY III	Cafeteria background noise	Memory recall (auditory), response time (visual)	117–133
T12: attended speaker II	Radio with traffic news background, speech from different room	Speech in speech, comprehension task (auditory)	134–146
T13: saliva sample	No background noise	Stress hormones (cortisol, alpha-amylase)	147–151
T14: d2-R	No background noise	Character scanning accuracy (visual)	152–161

OLSBY, OLERT and attended speaker were presented 2–3 times per session. All tests were performed in an aided and an unaided condition. When listening aided, in the second run of the OLERT (T10), the Speech signal was directly streamed to the participant's HAs using a TV connector. When listening unaided, the signal was presented via virtual TV loudspeakers. The d2-R, a visual attention task, was the only nonauditory test performed at the start and the end of all measures.

As shown in Table 1, salivary samples were integrated into the test procedure at four different time points: T1, T5, T9, and T13. To assess the overall effect of effortful listening on cognitive performance, the d2-R visual attention test was performed at the start and the end of the test session (see Table 1: T2 and T14). After each listening task, participants had to fill out a questionnaire regarding their effort needed to engage in the last task, and their current level of LRF after the completion of the last task.

The modified version of the dual-task paradigm by Hornsby (2013), the *OLSBY* (acronym OLSBY combines “Oldenburg” and “HornSBY”), was included in the test procedure at three points in time (see Table 1: T3, T7, and T11). An auditory vigilance task assessing RTs on target words, the *OLERT* (acronym OLERT is a combination of “Oldenburg” and “aLERT”), comparable to test paradigms used by Dinges et al. (1997) and described by Lieberman (2007), was implemented in the procedure (see Table 1: T4 and T10). Furthermore, an informationally complex task with switching target talkers as a multitalker speech-understanding scenario, the *CCOLSA*, as introduced by Heeren et al. (2022; *ConCurrent OLSA*), was included in the middle of the TCAD (see Table 1: T8).

EEG was recorded continuously during the whole test sequence, and the *Attended Speaker* test (see Table 1: T6 and T12) was specifically designed to investigate AAD. Due to technical problems, most of the EEG data was not suitable for analysis. Therefore, the EEG data can be found in the Appendix.

### Setup

**Figure 1.**
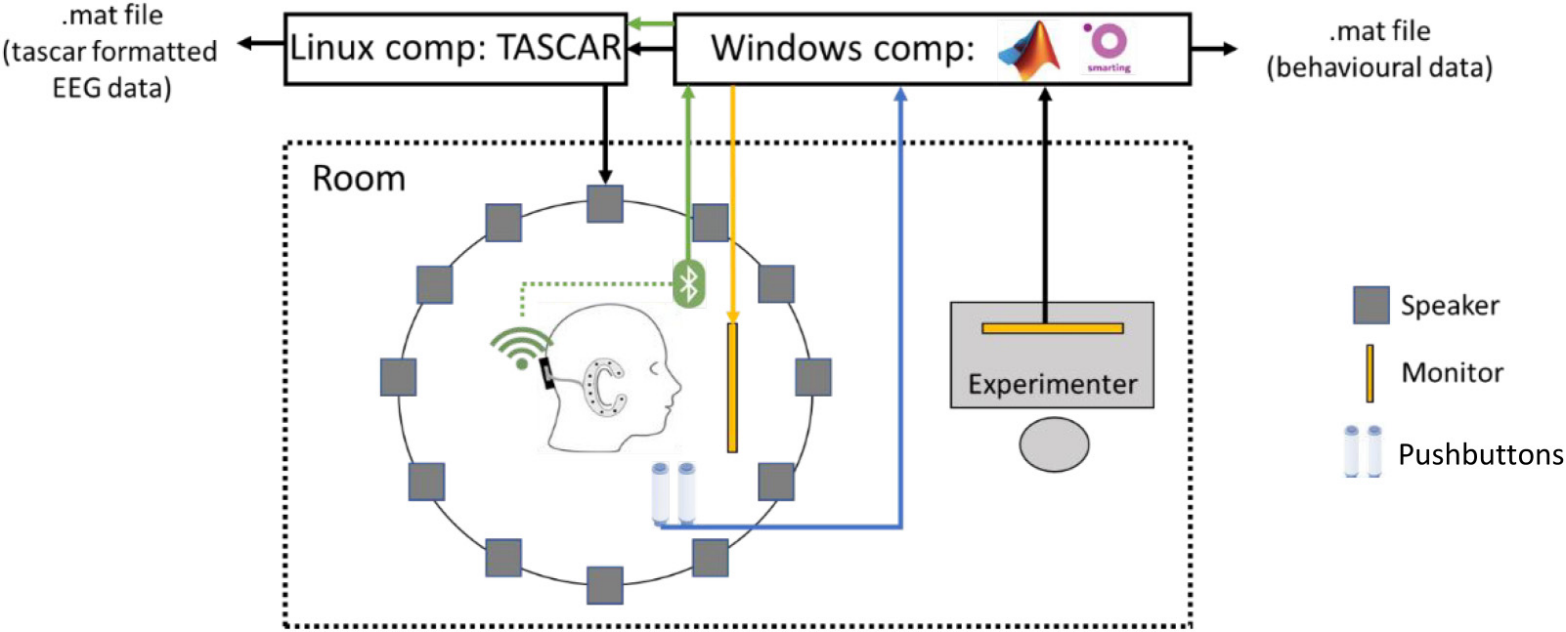
Experimental setup. Subjects were seated on a chair surrounded by a horizontal circle of loudspeakers. Two pushbuttons were handed to the subject and a mobile EEG-system was used. EEG data were sent to a Windows computer via Bluetooth and the data saved on a Linux computer. Behavioral data, such as reaction times, word recall, and so on was saved on the Windows computer. The Linux computer was used for scene playback and the Windows computer started the measurement scripts. A touchscreen monitor in front of the subject was used for vigilance testing (red flashing as trigger).

Measurements were conducted in two free-field labs of the Hörzentrum Oldenburg, Germany. Both measurement rooms had a size of approximately 18 m², reverberation times (T60) of approximately 0.2 s, and were equipped with an equivalent setup as shown in Figure 1. Sounds were presented using a horizontal loudspeaker array having 12 Genelec8030B loudspeakers, set up in 30° steps around a radius of 1.65 m. In front of a touchscreen in the center of the array was a rotatable chair, where the participants were seated. The virtual acoustic environments were created using TASCAR (Grimm et al., 2019). Background sounds were rendered in fifth-order Ambisonics. Target sounds were presented using nearest-speaker panning. On a second computer, experiments were controlled using MATLAB R2019a. Besides subject responses, neural brain activity was measured using a mobile EEG approach (see Appendix).

### The TCAD—Listening Task Paradigms

#### *OLSB*Y—Dual Task

The *OLSBY* is a dual-task paradigm similar to that used by Hornsby (2013), but combining Hornsby's approach with Oldenburg matrix test sentences. A cafeteria background noise (Grimm & Hohmann, 2019) was presented during all OLSBY sessions and the test was conducted three times (see Table 1). The test procedure comprised matrix sentences of the OLSA (Wagener et al., 1999) that were presented sequentially from the front loudspeaker at 0° Azimuth in a cafeteria background noise scenario play back from all 12 loudspeakers (see also Figure 1). The sentence matrix has the structure name–verb–number–adjective–object and contains 10 of each word class. Participants were instructed to look at a cross on the touch screen. The monitor highlighted red after a presentation of 4–10 sentences at a random point in time. The task was to (1) repeat the name and object of the last two sentences (e.g., “Peter, Auto; Kerstin, Sessel”; primary task) and (2) react as quickly as possible via pushbutton whenever the monitor highlighted red (secondary task). One measurement comprised 40 trials and took about 15 min. During the OLSA sequence, the pause between two sentences had a duration of 300 ms. Hornsby (2013) used presentation levels based on individual speech reception thresholds (SRT). Accordingly, the OLSA sentences were presented at an individual SRT of 50% correct responses plus 4 dB. These were measured in the training session for this particular speech material with the corresponding speech-shaped noise at 65 dB SPL (procedure: A1 by Brand & Kollmeier, 2002). The cafeteria noise (Grimm & Hohmann, 2019) during the OLSBY was presented at 67 dB SPL. RTs between the red screen trigger and the button press were evaluated, as well as the proportion of correct answers in the speech task.

#### *OLERT*—Vigilance Test

The *OLERT* paradigm is an auditory vigilance test (comparable to tests introduced by Pang et al., 2005 and Lieberman, 2007), based on the presentation of a novel as read by a male speaker. The test procedure is intended to measure the participant's alertness via the assessment of RTs. The test was performed twice per TCAD with different background noise distractors (see Table 1). Similar to a home environment, the acoustic scenarios of the OLERT were composed so as to mimic a breakfast situation or a stay in the living room. Therefore, a breakfast scene based on a diffuse kitchen background at 59 dB SPL, with a refrigerator, a clock and dish-washing sounds from a sink were presented during the first OLERT measurement. The target speech was presented via a virtual TV loudspeaker from 0° Azimuth at 62 dB SPL. During the second OLERT measurement, a living-room scene was presented from an adjacent kitchen, with a radio show reading the traffic news (presented from 270° azimuth at 59 dB SPL). When participants listened unaided, speech was presented via a virtual TV loudspeaker from 0° Azimuth at 62 dB SPL (identical to the first session). When testing was performed in an aided condition, the target speech was streamed directly via a TV connector at preference level. The target speech, a novel, contained two trigger words that occurred with a defined recurrence of 5 to 6 times per minute (total for both trigger words), at an overall length of 10 min. To minimize training effects, different sections of the novel were used each time the OLERT was performed. The number of target-word occurrences during the test was equal for all sections, while their length ranged from 609–708 s. The trigger words were chosen as the name of the protagonist “Harry” and the German personal pronoun “er.” Two pushbuttons were handed to the participant: The right button for the name of the protagonist and the left button for the personal pronoun. Whenever a trigger occurred, the participant had to press the correct pushbutton. The differentiation of the two trigger words implied the possibility of confusing trigger words and demanded a high concentration on the task. RTs between the beginning of the words and the button press were measured. A RT greater than 2 s was counted as a miss.

### Attended Speaker—Selective Attention Comprehension Task

The *Attended Speaker* test is a selective-attention comprehension task, performed in a homelike listening environment with a radio constantly presenting the traffic news in the same room and the target speech stream coming from a separate virtual room. The two competing sound streams (target vs. distractor) were thought to elicit selective attention effects in the EEG data, as described by Holtze et al., 2022. The target stream was the fairy tale “Zwerg Nase” (see also Mirkovic et al., 2016) read by a male speaker. Different sections were presented per trial. The target sound source was positioned behind a virtual wall, with a door at 270° azimuth enabling sound transmission from one room to another. As there was no direct sound path from the source to the listener, high frequencies were damped, and the sound impression was more reverberant. It was presented at a level of 60 dB SPL. The radio distractor was presented at 90° Azimuth at a level of 53 dB SPL. After a presentation of 10 min, participants were asked to answer 10 multiple-choice questions (one correct response and four distractors) about the fairy tale.

### *CCOLSA*—Multitalker Listening Test

The *CCOLSA, “*ConCurrent OLSA” test (Heeren et al., 2022), is a combined call-sign detection and speech-recognition paradigm based on three talkers that mimic a group conversation in a lively environment (in case of this study a public house). Therefore, three talkers alternately presented sentences of the OLSA (Wagener et al., 1999), while the public-house noise was played constantly. Each time a sentence started with the name “Kerstin” (call sign), the talker who said “Kerstin” was the new target talker until the next “Kerstin” occurred. The participants were instructed to orient towards the current target talker. When the current target talker was successfully identified, participants had to repeat the last words of all following sentences of the current target talker while ignoring the other two talkers. While the participants were engaged in the intelligibility task, they were constrained to remain attentive to the other two talkers, as sentences were still presented alternately from all talkers and the call sign would be spoken by a different talker after 2–5 sentences. The alternating sentence presentation was implemented with a partial overlap of 0.6 s. The three talkers had different voices: a male voice at −60° (M1), a female voice at 0° (F1) and a different male voice at 60° azimuth (M2). The public-house scene was presented at 68 dB SPL. To achieve an equal intelligibility of the three talkers, speech levels were established as described by Heeren et al. (2022) and amounted 68 dB SPL for talkers F1 and M2, and 67 dB SPL for talker M1. Word recognition of the target speaker was measured and analyzed.

### d2-R—Attention and Concentration Test

**Figure 2.**
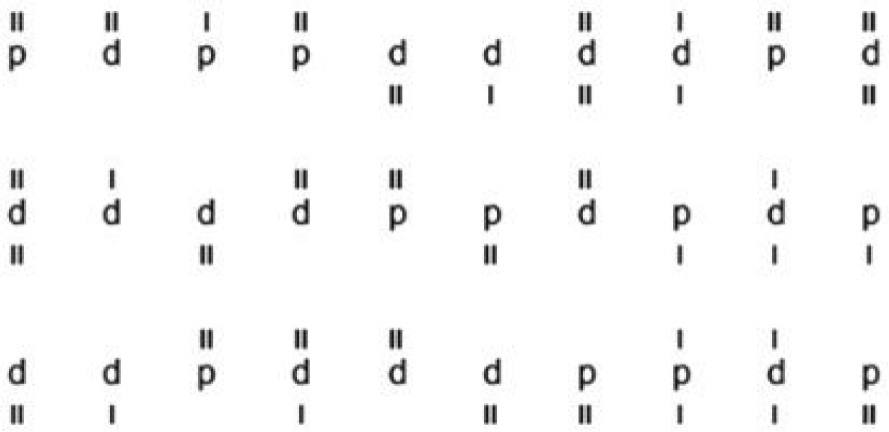
Example test sequence of the d2-R test.

The d2-R is a paper-and-pencil test for attention and concentration in which subjects were asked to mark targets (the letter “d” with two lines “II” above or below it) in rows of “d,” or “p” letters with one to four lines above or below them as shown in Figure 2 (Brickenkamp, 1962). The participant was asked to complete the task as fast and accurately as possible. The test consisted of 14 rows in total. For each row, the participant had 20 s before a switching to the next row, as instructed. As subjects were instructed to work as fast as possible, but without errors, the test stresses both speed and accuracy. The last marked target object (“d” with two lines “II”) in each row was used to calculate the number of processed targets (PT) by summing the number of targets found plus the number of targets which were overlooked (errors of omission [EO]) across rows. The PT score is a measure of processing speed without consideration of accuracy. To assess the overall concentration performance (CP), errors of commission (EC) are included by counting the marked distractors (a “d” with the wrong number of dashes or any instance of “p”). The CP score was then calculated as follows: CP = PT–EO–EC. To ensure optimal visibility of the visual stimuli, participants wore their glasses and confirmed their ability to perceive the stimuli clearly.

### Subjective Evaluation of Listening Effort and Fatigue

The momentary states of LE and LRF were monitored during the complete procedure by noting subjective ratings. Subjects were given a sheet with two 10-unit-scales to judge both LE and LRF after each listening task (OLERT, CCOLSA, Attended Speaker, and OLSBY). Subjects had to rate the concentration needed to perform the last task (“How much did you have to concentrate to understand the speaker?”) on a scale from 0 (*did not have to concentrate*) to 10 (*had to concentrate very hard*) and had to rate their current level of LRF (“How mentally exhausted or tired do you feel after the task?”) on a scale from 0 (*not exhausted*) to 10 (*very exhausted)*. Changes in subjective momentary LE and LRF throughout the TCAD were analyzed.

### Physiological Assessment

For hormone sampling, Salivettes from Sarstedt AG & Co. KG containing cotton swabs were used. Subjects were instructed to insert the cotton swabs directly into their mouth and chew on them for at least 2 min on both sides of the mouth so that the swabs could be filled with saliva. They then put the swabs directly into the Salivettes without touching them. Next, the Salivette was labeled with the subject's ID, the date and exact time, and placed immediately in a freezer to ensure temporal stability. Samples were sent in a polystyrene package to Dresden LabService GmbH for analysis of cortisol and alpha-amylase concentrations. Since cortisol levels vary with the circadian cycle, as a baseline to eliminate this as a possible confounding variable, saliva samples of all participants were also taken on nontest days at the same time sampling occurred during the TCAD. Participants were instructed to perform the sampling at the appropriate times at home on their own. The exact start time was carefully controlled on each test day and for the baseline assessment. Except for one subject, who started 1 h earlier on the second visit, no deviations occurred. For the other 19 subjects, the mean difference between the start time on Days 1 and 2 was 5 min ( ± 3 min). These baseline measurements served as a correction, to make short-term effects in stress hormones visible. Changes in cortisol (nmol/l) and alpha-amylase levels (U/ml) were analyzed.

### Test Procedure

The measurements were performed in three sessions. Session one involved a screening for subsequent testing and training, allowing participants to become acquainted with the different listening tests and hearing situations. The intention was to ensure that participants were both able to perform the tests and become familiar with them, to minimize training effects before starting the main data collection. All participants were instructed to use their HAs during training. Two participants were not able to perform pretesting properly and were eventually replaced. In session two and three, participants completed the whole test sequence in either unaided or aided conditions, with randomized test order across participants. The lab room was not changed during an ongoing measurement. No information on task progress was provided to the participants. Participants were instructed to ask for breaks in between tests if necessary. However, all subjects were able to participate without breaks. Sessions one, two, and three were scheduled on different days, but at the same time of day during the morning. 

### Participants and Hearing-Aid Fittings

**Figure 3.**
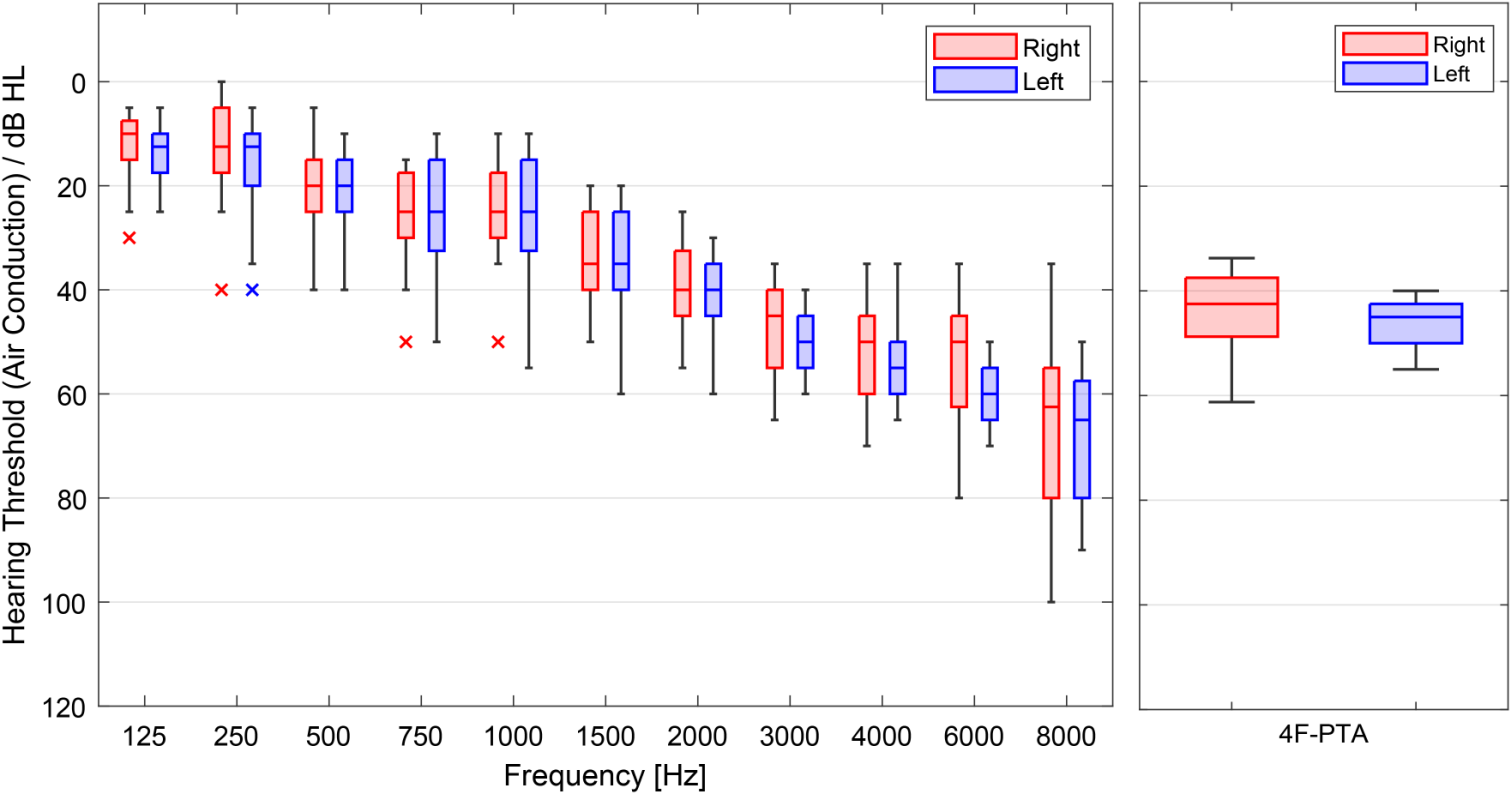
Boxplots of audiometric data of all 20 subjects for frequencies from 125 to 8000 Hz (left panel) and boxplots of the four frequency puretone average across all subjects (right panel). Boxplots show medians, quartiles, minima, maxima, and outliers.

Participants were 20 experienced HA users (10 female/10 male) with mild to moderate sensorineural hearing loss (Grade 1 and 2 according to WHO, 2021) and had no history of neurological disorders. Participants of the female group had a median age of 73.5 years (minimum = 57 years, maximum = 83 years), and the median of the male group was 68 years (minimum = 64 years, maximum 76 years). The left panel of Figure 3 shows the median hearing loss across all participants for right and left ears for all tested frequencies from 125 to 8000 Hz. The right panel shows the median 4F-PTA (“four-frequency pure-tone average”: pure-tone audiogram averaged across 0.5, 1, 2, and 4 kHz) across all participants for the right and left ear: The median 4-FPTA of the right ear was 43 dB HL, with one participant's 4F-PTA at 34 dB HL (minimum) and one at 61 dB HL (maximum). The left-ear median 4F-PTA was 45 dB HL, with a minimum of 40 dB HL and a maximum of 55 dB HL. Except for one participant, the difference between the left and right ear within the 4F-PTA range did not exceed 15 dB. The majority of participants (18/20) had a 4F-PTA right-left-difference of less than 10 dB. The remaining two participants had a difference of 10 and 15 dB between right and left 4F-PTA.

Participant's cognitive functions were assessed using a standardized screening test for dementia. The DemTect tests cognitive abilities such as memory, vocabulary, and attention in several short tests. This screening test is designed to detect cognitive dysfunction, particularly in the early stages of Alzheimer's disease and mild cognitive impairment (MCI), and has been validated as a sensitive tool for identifying patients with MCI and early dementia (Demtect, Kalbe et al., 2004). The test can be used to decide whether cognitive performance is adequate for age within about 10 min. Scores indicating normal cognitive functioning are between 13 and 18 points, and mild cognitive impairment may be present if scores are below 12. If one of the participants had scored less than nine points (suspicion of dementia), he or she would have been informed about the result in an extra information session in which they would have been advised to have a diagnostic assessment in a special clinic. No subject had a score of less than 12 points, however. The mean score across all subjects was 16 with a standard deviation (*SD*) of 2.

All participants were fitted binaurally with Phonak Audéo M90-312 HAs programmed at default settings for their hearing loss (including activation of adaptive parameters for each participant and frequency compression if relevant for their hearing profile). Coupling to the ear canal was achieved by closed domes. Study participation was compensated with 12€/h. All participants were briefed about the experimental procedure and completed a declaration of consent. Ethics were approved by the ethics committee (“Kommission für Forschungsfolgenabschätzung und Ethik”) of the Carl von Ossietzky University in Oldenburg, Germany (Drs.EK/2019/032, Drs.EK/2019/019).

## Results

### Analysis

Subjective data were analyzed using a multivariate General Linear Model (GLM) with repeated measures to assess the impact of HA provision on perceived LE and LRF, and subsequent post hoc testing was carried out using Wilcoxon-signed-rank (WSR) tests. The comparison of the behavioral data (word recognition and RTs) in the two test conditions was performed using one-sided *t*-tests. The physiological data were analyzed using a repeated measures GLM model and the data of the visual attention test (d2-R) was analyzed using a repeated measures ANOVA for pre- and post-TCAD comparison. For illustrational purposes, statistical outliers have been removed from all boxplots. Subjective and objective assessments of LE and LRF were evaluated for all 20 participants.

### Subjective Assessment

**Figure 4.**
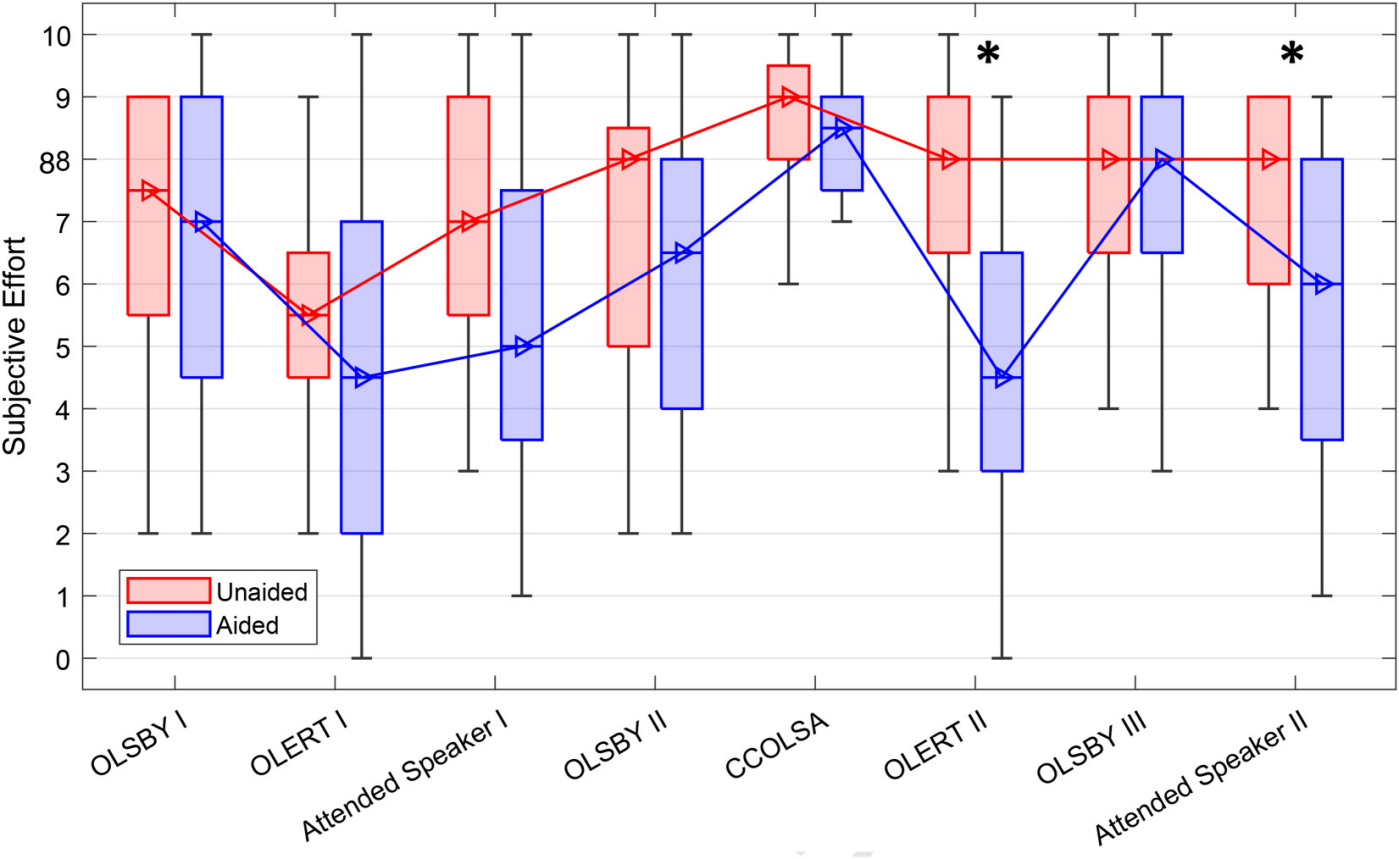
Subjective ratings of concentration (LE) during the TCAD. Boxplots show medians, quartiles, minima, and maxima. Significant differences between aided and unaided were found for OLERT and Attended Speaker using the WSR test (*p < .05).

Subjective ratings of LE on tasks across the TCAD are plotted in Figure 4 for both the unaided (red boxplots) and aided (blue boxplots) conditions, where a rating of 0 refers to no effort required and 10 corresponds to maximum effort required to follow the speaker. Overall, measures of LE were high across conditions; however, data reveal a trend of lower ratings in the aided versus unaided condition, indicating the need to invest less effort through the provision of HAs. The multivariate repeated measures GLM showed a main effect of task, *F*(7, 133) = 9.739, *p* < .001, $\eta_{p}^{2}$ = .339), provision, *F*(1, 19) = 15.69, *p* < .001, $\eta_{p}^{2}$ = .452), as well as an interaction of HA provision and task, *F*(7, 133) = 3.077, *p* < .01, $\eta_{p}^{2}$ = .139. Significant differences between aided versus unaided performance were observed for the second OLERT task (Bonferroni correction, WSR, *p* = .02) and the second Attended Speaker task (Bonferroni correction, WSR, *p* = .02).

**Figure 5.**
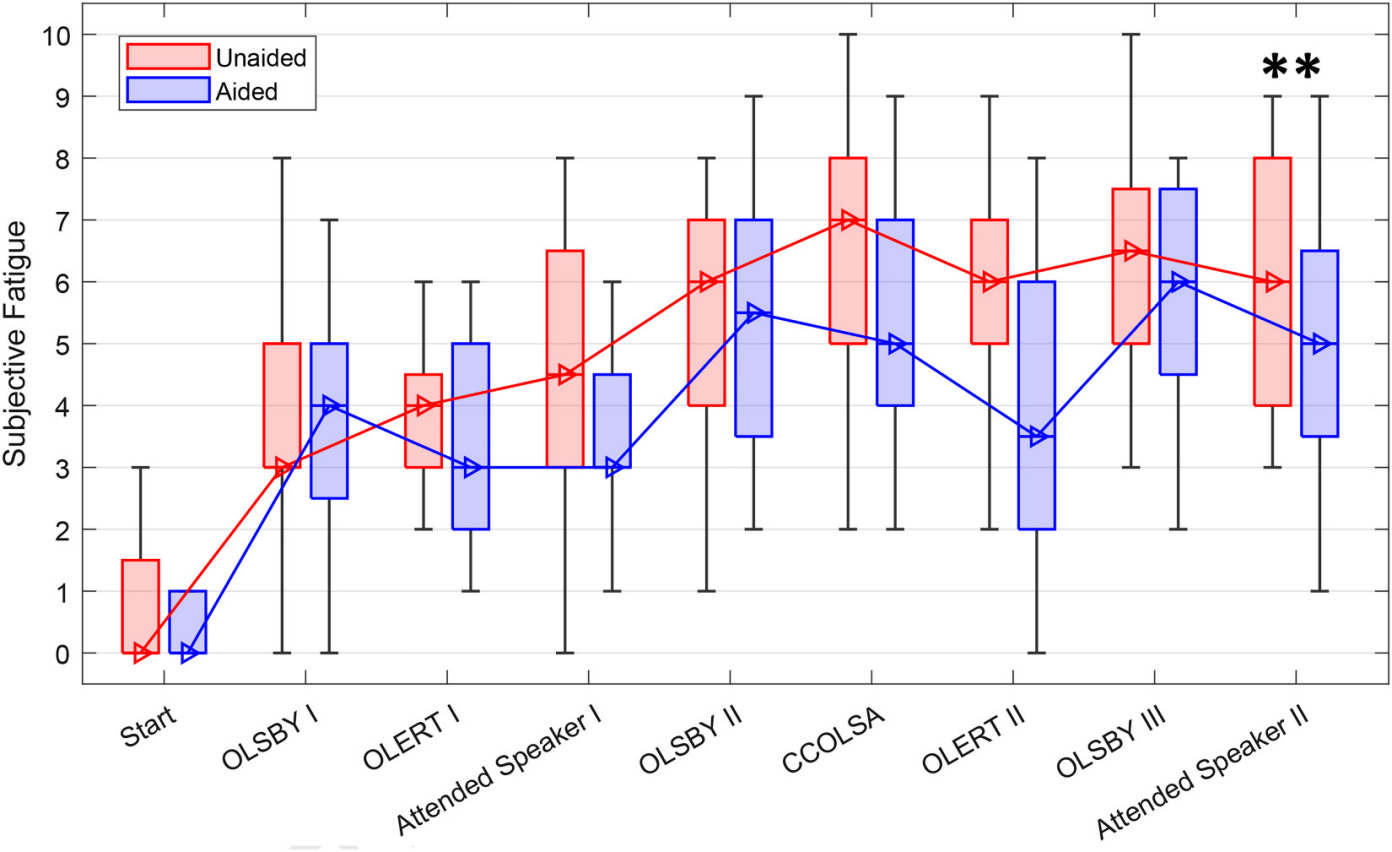
Subjective ratings of LRF during the TCAD. Boxplots show medians, quartiles, minima, and maxima. Significant differences were found for the Attended Speaker using WSR test (**p < .01).

Subjective ratings of LRF on tasks across the TCAD are plotted in Figure 5 for both the unaided (red boxplots) and aided (blue boxplots) conditions. The “Start” condition shows LRF before the start of the TCAD and serves as a baseline measure. LRF ratings increased throughout the TCAD in both the aided and unaided conditions. The multivariate repeated measures GLM showed a main effect of hearing aid provision, *F*(1, 19) = 5.311, *p* < .05, $\eta_{p}^{2}$ = .218, task, *F*(7, 133) = 39.31, *p* < .001, $\eta_{p}^{2}$ = .443, as well as an interaction between HA provision and the task, *F*(7, 128) = 3.079, *p* < .01, $\eta_{p}^{2}$ = .139. The largest aided-unaided difference in rated LRF was observed in the second OLERT task (TV connector usage), with a median LRF score of 3.5 versus 6, respectively, reaching significance (Bonferroni correction, WSR, *p* = .003).

**Figure 6.**
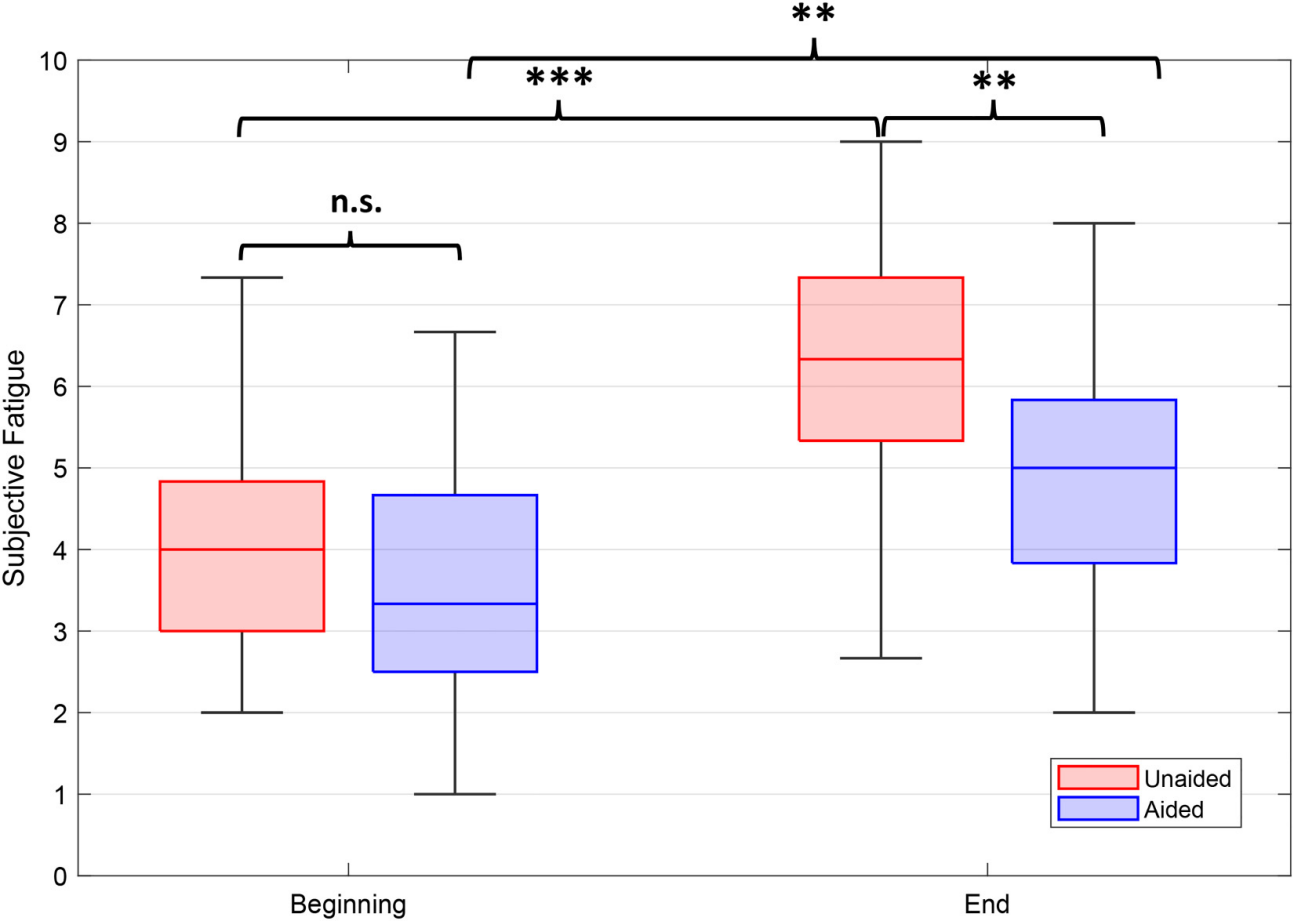
Averaged LRF ratings over the three tasks OLERT, OLSBY, and attended speaker, calculated for the beginning and the end of the TCAD. Boxplots show medians, quartiles, minima, and maxima. Differences were analyzed using a WSR test and significant differences are indicated (n.s.: not significant; **p < .01; ***p < .001).

Following the significant provision by task interaction of the GLM model, we calculated an average score across the first three and the last three listening tasks. Note that these are same test paradigms in a different presentation order and in case of OLERT with differing acoustical scenario as well as different novel excerpts (see test descriptions and Table 1 in Methods section). WSR test was performed to compare differences between the aided and unaided condition, as well as between the beginning and the end under both conditions. Figure 6 illustrates the differences between ratings at the beginning and at the end of the TCAD. Subjective LRF was not significantly different between the aided and unaided test condition at the beginning of the TCAD. However, significant differences in subjective ratings of LRF were observed between the start and the end of the TCAD in both the unaided (*p* < .001) and, to a lesser, albeit significant, level in the aided condition (*p* < .01). Comparing LRF scores of both listening conditions at the end of the TCAD, a highly significant difference was observed (*p* < .01), with an overall higher LRF score in the unaided condition.

### Behavioral Assessment

**Figure 7.**
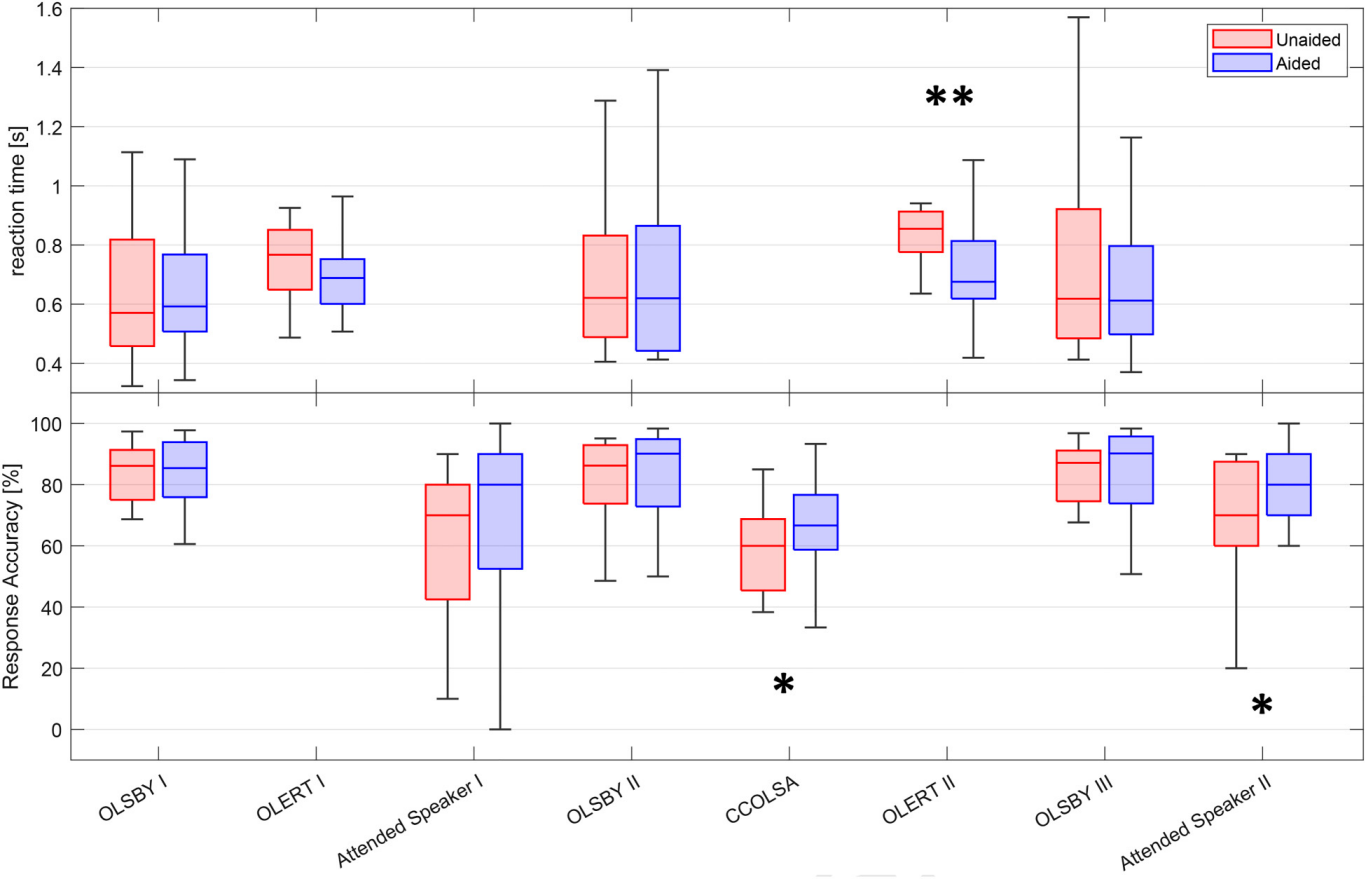
Rts and word-recall accuracy during the TCAD. Boxplots show medians, quartiles, minima, and maxima. Differences were analyzed using one-sided *t*-tests. Significant differences between aided and unaided are indicated in the figure (*p <.05, **p <.01).

Figure 7 shows the behavioral assessment results obtained during the TCAD, with RT plotted on the upper-part and word-recall accuracy plotted on the lower aspect of the figure.

During the three sequences of the OLSBY dual paradigm task, median RTs ranged between 0.57 to 0.62 s, and median response accuracy ranged between 85%–90% across the aided and unaided conditions. Differences in RTs and response accuracies were statistically analyzed using one-sided *t*-tests, with no differences found in RTs between aided vs. unaided conditions across any of these runs. Similarly, no significant differences were observed in response accuracy between the unaided and aided conditions across the three runs.

For the OLERT tests, median RTs ranged between 0.69 and 0.86 s across the two test sequences. For the first OLERT, the one-sided *t*-test did not reveal statistical differences between aided and unaided testing (*p* = .021). The second OLERT trial (TV connector usage) led to a median reaction time of 0.68 s in the aided condition versus 0.86 s in the unaided condition. The difference was shown to be statistically significant (*t*-test, *p* = .008), suggesting an improvement in LE when streaming the speech signal directly into the HAs.

In the first sequence of the Attended Speaker test, speech was presented from the room next door, and median correct word responses of 70% and 80% were observed in the unaided and aided conditions, respectively, but this small improvement was not statistically significant (*t*-test, *p* = .122). In contrast, for the second sequence, whilst the same median correct word recognition scores of 70% and 80% were observed in the unaided versus unaided condition, respectively, the benefit derived from HA usage at the end of the TCAD was statistically different (*t*-test, *p* = .019).

For the CCOLSA multitalker paradigm, median correct word recognition scores were 62% versus 67% in the unaided versus aided condition, and this difference reached the level of significance (*t*-test, *p* = .017), implying a benefit of HA use in this simulated group-like communication situation.

### Physiological Assessment

**Figure 8.**
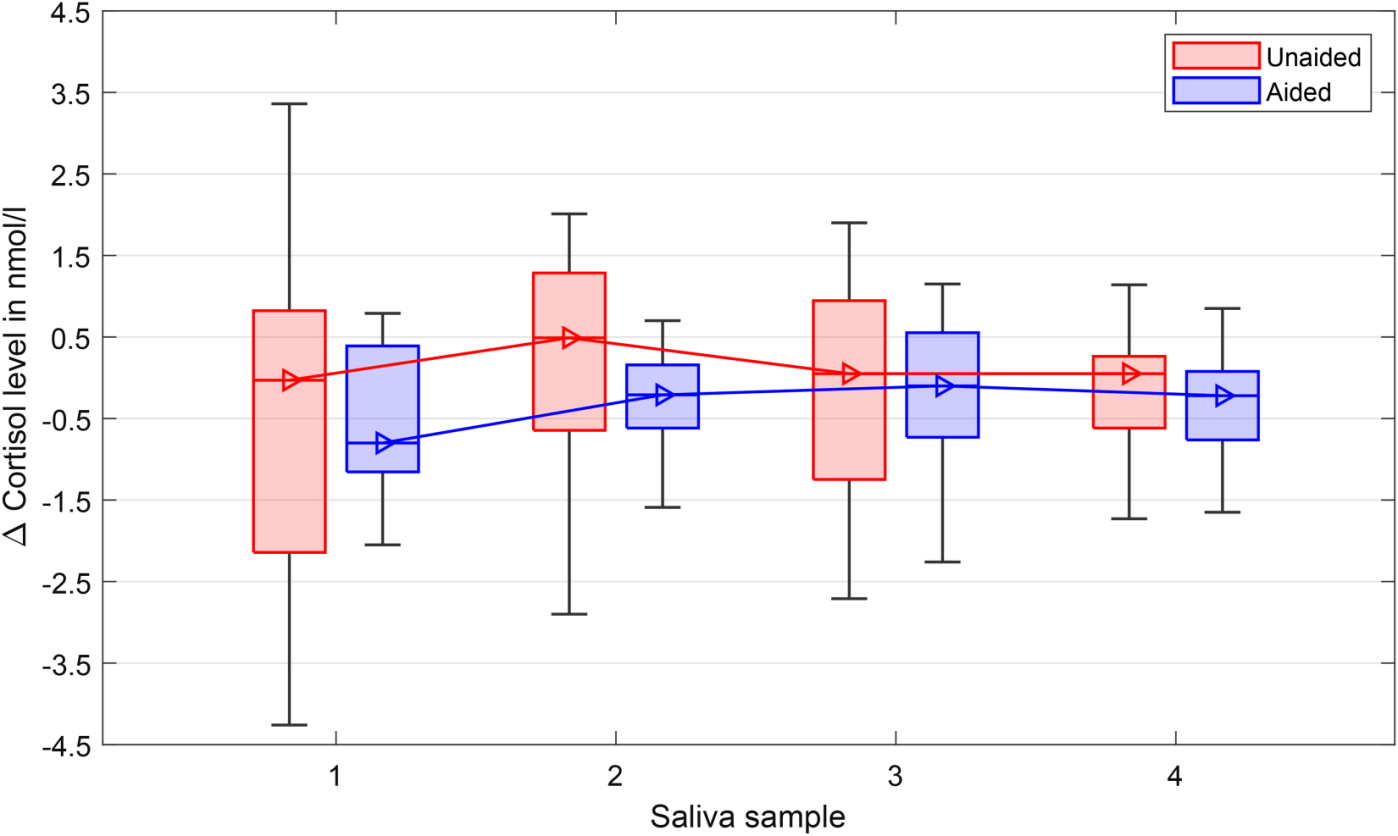
Cortisol levels over the four time points during the TCAD. Boxplots show medians, quartiles, minima, and maxima. Baselines (saliva samples at home at the same time points) were subtracted to avoid any influence of the typical circadian cycle of the cortisol levels.

Stress hormones were baseline corrected by subtracting cortisol and alpha-amylase baseline measurements from the home measurement from those obtained during test sessions (see Methods section “Physiological Assessment”). Figure 8 shows the median cortisol levels, measured over four time points T1, T5, T9, T14 as seen in Table 1 (Methods section). Data from three subjects were excluded, because their cortisol levels were much higher compared to the rest (above the mean plus twice the standard deviation). There is a trend of an increase in cortisol levels from timepoint one to two, with slightly lower overall cortisol levels in the aided condition. However a repeated measures GLM model established that there was no statistical main effect regarding hearing aid provision during TCAD over the four measurement points, *F*(1, 17) = 0.86, *p* = .37, $\eta_{p}^{2}$ = .05). Furthermore, no significant main effect of cortisol was observed, *F*(3, 51) = 0.60, *p* = .62, $\eta_{p}^{2}$ = .03. The interaction measurement time and HA provision was also not significant, *F*(3, 51) = 0.80, *p* = .45, $\eta_{p}^{2}$ = .05.

**Figure 9.**
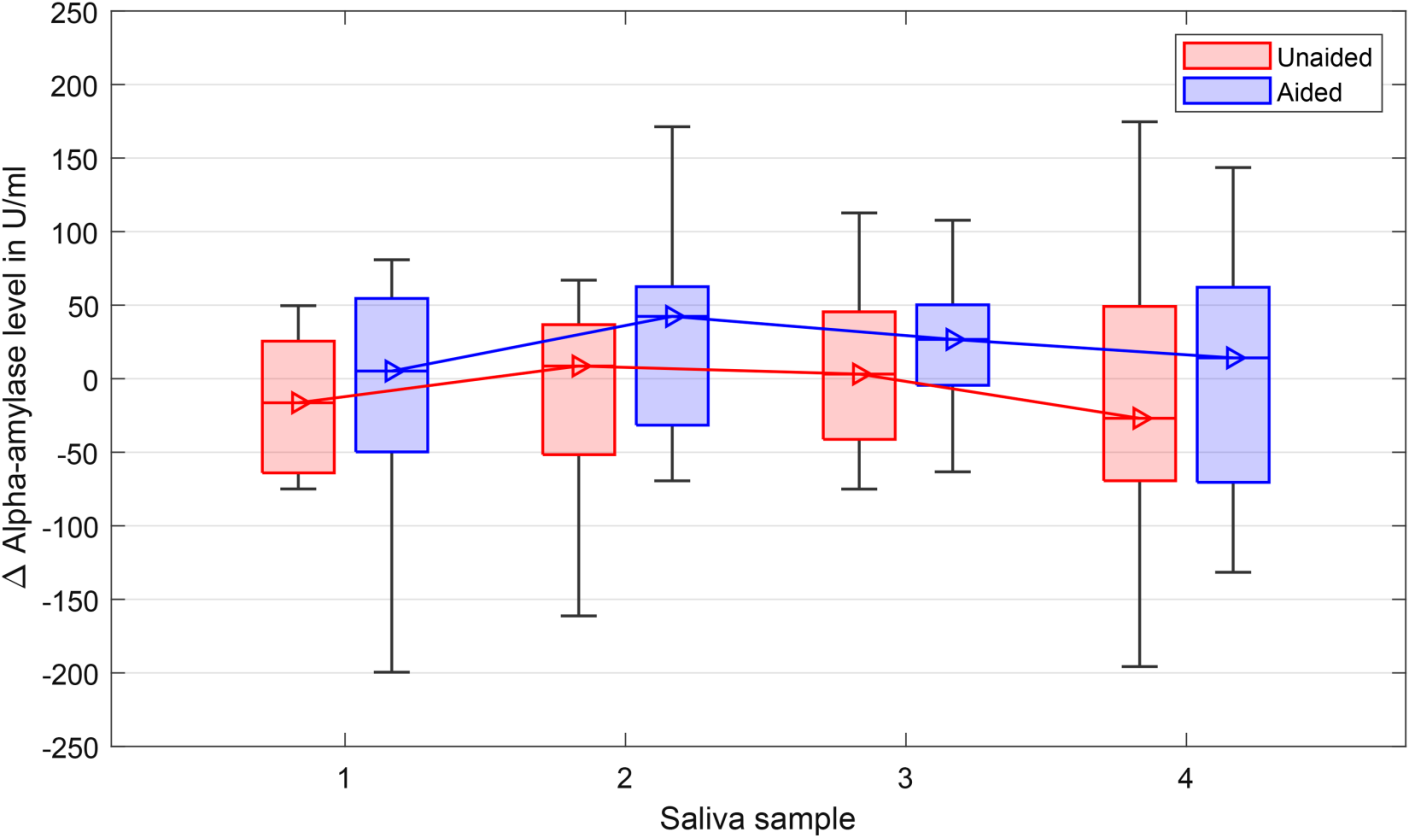
Alpha-amylase levels over the 4 time points during the TCAD. Boxplots show medians, quartiles, minima, and maxima. Baselines (saliva samples at home at the same time points) were subtracted to avoid influence of the typical circadian cycle of the amylase levels.

Figure 9 shows the median alpha-amylase levels at the same four time points T1, T5, T9 and T14. Data from 3 subjects were excluded because their amylase levels were much higher compared to the rest (above the mean plus twice the standard deviation). Consistent with cortisol levels, there was a trend of increasing alpha-amylase levels from time point one to two. Using the repeated-measures GLM model, no significant effects neither for the HA provision, *F*(1, 13) = 0.87, *p* = .37, $\eta_{p}^{2}$ = .06, and measurement points, *F*(3, 39) = 1.43, *p* = .25, $\eta_{p}^{2}$ = .09, nor for the interaction effect of HA provision by measurement points, *F*(3, 39) = 1.08, *p* = .38, $\eta_{p}^{2}$ = .07, were observed.

### d2-R—Attention and Concentration Test

**Figure 10.**
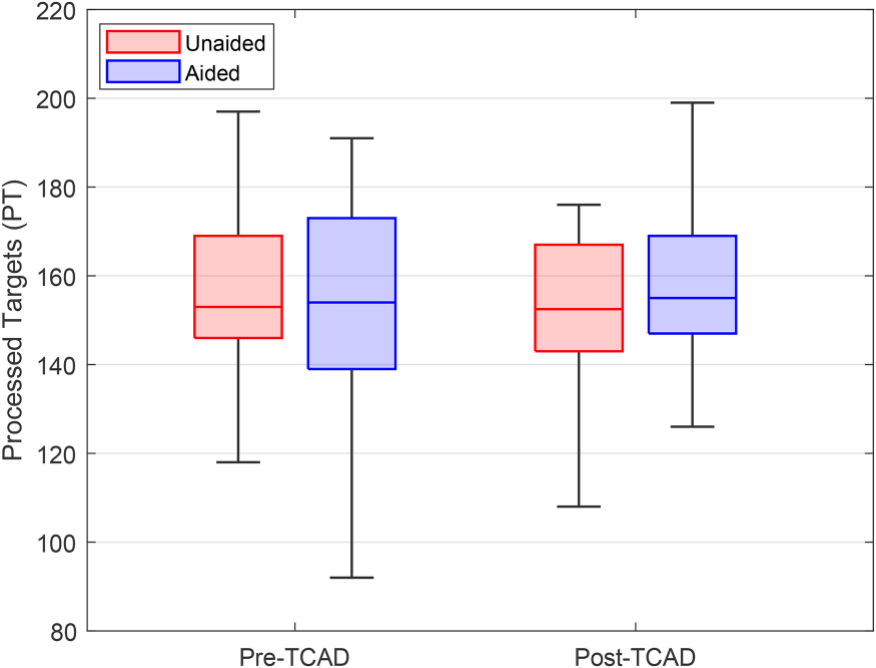
Comparison of the processed targets (PT) pre- and post-TCAD. Boxplots show medians, quartiles, minima, and maxima.

**Figure 11.**
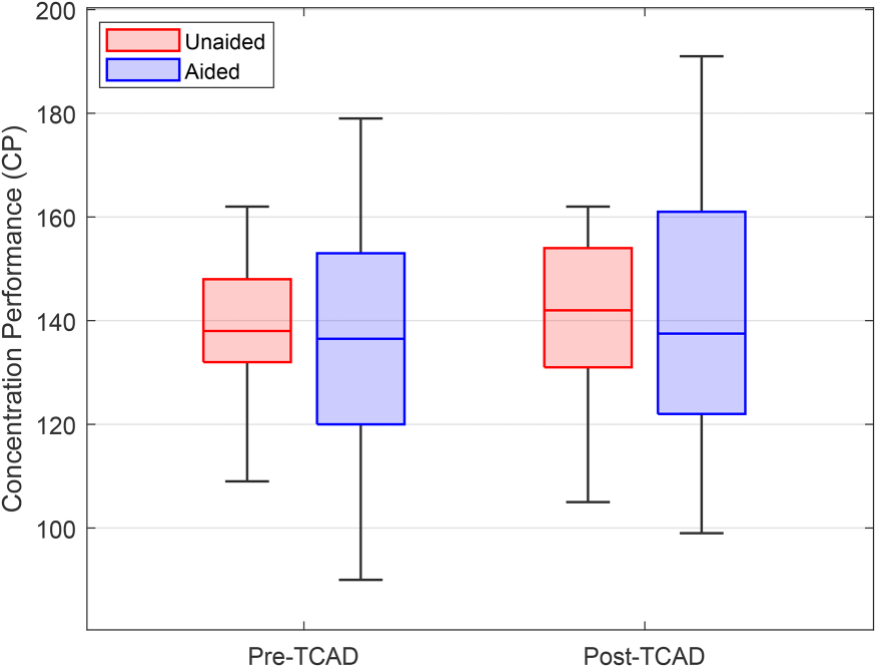
Comparison of the concentration performance (CP) pre- and post-TCAD. Boxplots show medians, quartiles, minima, and maxima.

Figure 10 shows the median in d2-R PT pre- and post-TCAD and Figure 11 the median CP. Similar to the analysis of the physiological data, subjects with errors above the mean plus twice the standard deviation were excluded from the analysis. It can be assumed that subjects with many EC completed the task with a focus just on speed, which could have biased the outcome (Brickenkamp et al., 2010). Two subjects fulfilled the defined criterion and were eventually excluded from the analysis, which therefore only included the remaining 18 participants.

In the domain of PT (Figure 10), a significant interaction between HA provision and time was found using a repeated measures ANOVA, *F*(1, 1) = 4.45, mean square (MS) = 206.72, *p* = .042. Corrections for sphericity were applied, with Greenhouse–Geisser and Huynh-Feldt adjustments yielding *p*-values of .042, supporting the significance of the interaction. Paired *t*-tests were conducted for pre- vs. post-TCAD for each HA condition separately as well as between aided and unaided. However, the subsequent post hoc testing did not reveal any significant differences. To allow a direct comparison of cognitive functioning before and after the TCAD, differences in PT were calculated by subtracting the post- from pre-TCAD measures intra-individually. Therefore, a positive value would indicate an improvement and a negative value a deterioration. The mean post- to predifferences in the unaided condition were −2 (*SD* = 11) score points. In the aided condition, mean score points were +4 (*SD* = 7). These two differences were statistically significant (*t*-test, *p* = .045).

Figure 11 shows the median of the d2-R CP pre- and post-TCAD. In the analysis, no significant effects were found using a repeated measures ANOVA.

## Discussion

This study explored the impact of HAs use on LE and LRF using a prolonged test-sequence, the TCAD, reflecting daily-life acoustical listening scenarios and aiming for a degree of ecological validity (Keidser et al., 2020) using a diverse set of metrics such as subjective ratings of LE and LRF, as well as behavioral and physiological measures.

The high subjective ratings of LE that were observed in both the unaided and aided conditions indicate that participants had to concentrate intensely during the TCAD. Comparing aided and unaided testing, lower LE ratings were observed in the aided condition for two tests, the second run of the OLERT and the second run of the Attended Speaker. This is in accordance with previous research, showing that HAs can reduce LE (e.g., Bentler et al., 2008; Holman et al., 2019; Noble & Gatehouse, 2006; Winneke et al., 2020). This observation was confirmed by the GLM analysis, which revealed an interaction between HA provision and task.

LRF ratings increased substantially throughout the TCAD for aided as well as for unaided testing. This confirms hypothesis one that LRF ratings will increase throughout the TCAD. It is in accordance with interview results obtained by Holman et al. (2019), where participants emphasized that fatigue develops after experiencing demanding listening situations. Similarly, Burke and Naylor (2020) reported that both normal hearing and hearing-impaired participants became more and more fatigued throughout the day after experiencing daily-life listening situations. The observed high levels in self-rated effort can be thought of as a compensatory mechanism for sustaining task performance during the continuously high workload throughout the TCAD (Hockey, 2011) and as all participants were experienced HA users, regularly participating in studies, and paid for their participation, we assume their motivational levels to engage in the test procedure were high. Therefore, the increase in self-rated LRF is not interpreted as a sign of a loss of interest in the task or a loss of motivation. In this context, the peak in subjective LE towards the middle of the TCAD (CCOLSA) with simultaneously high LRF ratings is most likely a cognitive adaptation process, necessary to meet the demands of the specific listening task. Following this interpretation, the increasing self-rated LRF was a result of the high, sustained effort and not just a byproduct, but a consequence of the depletion of cognitive resources (Pichora-Fuller et al., 2016). This increase in LRF became particularly evident when comparing the averaged ratings from the beginning and the end of the TCAD, showing a significant increase for both test conditions, but significantly lower LRF ratings at the end of the TCAD when testing aided. Correspondingly, the GLM analysis showed a main effect of HA provision as well as an interaction between HA provision and task. Post hoc, a significant difference in LRF with less self-rated LRF when using HAs was also found for the second run of the Attended Speaker task by the end of the TCAD. Taken together, these findings confirm hypothesis five, that is, that HAs can reduce LRF. Furthermore, LRF findings are consistent with the study from Holman et al. (2021b) who observed an improvement of LRF in daily life when using HAs in comparison to a control group with hearing loss and no HAs. A large data analysis from the EuroTrak survey by Bisgaard et al. in 2017 goes in a similar direction. Evaluating a data set of over 10,000 people with self-reported hearing impairment, they found that hearing aid users were 14.5% less exhausted at the end of the day compared with nonusers with similar hearing losses. Although the findings by Holman et al. (2021b) and Bisgaard and Ruf (2017) support our findings, the comparability between the different studies remains low.

While the subjective results imply that the hearing test sequence within the TCAD required LE that accumulated to sensations of LRF over time, identifying the onset of LRF resulting from prolonged LE remains unclear and the onset of fatigue may vary across individuals. Indeed, Hornsby et al. (2016) emphasized the influence of subject- and task characteristics on the development of fatigue. Furthermore, an individual's motivation and/or state of mind may also play a role; for example, someone in a positive state of mind might be prone to underestimating the effort needed to engage in a task and the resulting fatigue, whereas someone in a negative state of mind might systematically overestimate effort and fatigue (McGarrigle et al., 2014). In fact, declining motivation could even be a marker for increasing fatigue (Hornsby et al., 2016). The level of interest in the task has also been shown to be linked to self-rated levels of fatigue; specifically, those interested in a task tend to report lower levels of fatigue (Milyavskaya et al., 2021). The connection between interest, motivation, and self-perceived effort and fatigue might partially explain the variability in the obtained subjective ratings. However, a meta-analysis by Carolan et al., 2022 suggests that motivational factors only have a small-to-medium effect on LE and with the randomized cross-over study design, we aimed to reduce motivational effects between test conditions. To gain insights into the relationship between LE, motivation, and emerging LRF, in future studies the participant's motivation should be systematically assessed. For this purpose, after the completion of each TCAD task, participants could fill out a questionnaire about their self-rated motivation to further engage in testing.

Advanced HA features, such as directional microphone techniques have been shown to impact LE (e.g., Winneke et al., 2020), but as reported in the study of Hornsby in 2013, techniques such as directional processing and digital noise-reduction algorithms might not affect LRF. The HAs in this study were all fitted in default settings. This means that the HAs adaptively controlled whether advanced features, e.g., for noise reduction, were on or off. This applies to all modern HAs, and is the normal setting in everyday life and therefore the most ecologically valid way to test the influence of HAs on LE and LRF. However, a possible reduction in LE and LRF due to these features was not systematically tested in this study and remains the subject of future research. Though all participants were experienced HA users, and HAs were fitted individually, we did not provide a specific acclimatization phase prior to testing. This might in some cases have led to a small performance decrement in comparison to usage of their own HAs. However, the effects of not using one's own hearing aid are expected to be smaller compared to the effects observed in aided vs. unaided testing. It should also be noted in this context that more profound hearing losses would in principle have led to greater effects in some tests. However, as the tests were intended to be performed without further modifications, both aided and unaided, a mild to moderate hearing loss was selected.

Whereas the behavioral tests in the TCAD were intended to accumulate effort and induce fatigue across the test session, the OLSBY was specifically included to try and replicate the findings of Hornsby (2013). The testing of Hornsby (2013) took 50 to 60 min and was only interrupted by the participant responding to one of the tasks. Additionally, baseline RTs were measured in a “RTs only” single task. He observed an increment in RT over the duration of the task for unaided listening compared to stable RTs in the aided condition. Subjective ratings of fatigue and attentiveness also increased significantly after the task was completed, but differences between unaided and aided ratings were not observed. In contrast to the findings of Hornsby (2013), in the current work no increases in RTs were observed towards the end of the TCAD. An obstacle in replicating his findings may have been a ceiling effect in the primary task (word recall). The response accuracy was above 80% for all runs and all conditions, suggesting that this task might not have been sufficiently demanding to observe changes on the secondary task (RTs). Moreover, the task changes during the TCAD potentially caused goal changes in the subjects’ motivation, which could have led to a reset of effort and a reduction of fatigue (Hockey, 2011). Significantly improved response accuracy was observed in both the CCOLSA and the Attended Speaker paradigms when participants used HAs. Furthermore, significantly reduced RTs were observed in the second OLERT run in the aided condition, most likely due to enhanced listening provided by the direct streaming of the target signal via the TV connector (Picou & Ricketts, 2011). This is consistent with previous studies that showed improved performance with wireless signal streaming; for example Sjolander et al. (2009) reported that the use of a TV adapter system can improve television understanding for HA users. However, none of the behavioral data shows a significant performance decrement that reflects LRF. Consequently, hypothesis two (“Behavioral measures, such as RTs and word recognition, will decline during the TCAD.”) was not confirmed.

The examination of the stress level measured with the hormones cortisol and alpha-amylase showed no statistically significant differences over the time course of the TCAD, which rejects hypothesis four (“Stress hormone levels will increase with ongoing test duration.”). To assess cortisol concentration, the optimal timing of sampling is approximately 20–30 min after the onset of the stressor. Alpha-amylase levels are expected to peak earlier (5–10 min after onset, see discussion in Kramer et al., 2016 and Engert et al., 2011). As the last sample was taken just a few minutes after the last test, the timing was not ideal for cortisol, but for alpha-amylase concentration it was as recommended. However, due to the length of the TCAD of more than 120 min, some listening-related effects were also expected for cortisol levels. A potential confounder for cortisol and alpha-amylase levels is that food and drinks might interfere with the concentration (Bhattarai et al., 2018). Often subjects ask for a coffee before a study starts, but this was not allowed in the case of the TCAD study. However, food intake of the subjects was not controlled, but since it took time to arrive and prepare at the test facilities, it is assumed that the last meal was at least 30 min past, which is in agreement with the “Compliance with Saliva Collection Protocol in Healthy Volunteers” protocol by Bhattarai et al. (2018).

Reviewing the stress-hormone results on a descriptive level, a small increase in cortisol levels and a decrease of alpha-amylase was observed across the TCAD in the unaided condition, especially from the first to the second sampling point. However, the effect was small and not significant. The variations in amylase of  < 30 U/ml in the current study were much lower than the variations of > 100 U/ml reported by Nater et al. (2005) for their psychosocial-stress paradigm. Similarly, the descriptive changes in cortisol were also lower compared to the differences shown by Bess et al. (2016). They investigated the diurnal changes of cortisol in hearing-impaired and normal-hearing children and showed an increased cortisol awakening response of about 2 nmol/ml compared to normal-hearing children. Whereas the current study investigated the cortisol response to specific task-related demands, that is, the TCAD, Bess et al. (2016) investigated the sustained or long-term fatigue due to hearing impairment.

Francis and Love (2019) discussed the effect of LE on cortisol (Chapter 3.2.5 Neurochemistry) and stated that “…there has not yet been strong experimental evidence supporting a clear relationship between short-term listening challenges and increased levels of stress-related neurochemical responses.” This is in line with a pilot study by Kramer et al. (2016), who investigated the effect of a speech-recognition task on cortisol, chromogranin, and the pupillary response in normal-hearing and hearing-impaired listeners. Their test session took less than 1 hour, and they found no effect on cortisol concentration. Similar findings were reported by Zekveld et al. (2019). They studied the effect of a social-evaluative threat on speech recognition, subjectively-experienced hearing difficulties, and the two biomarkers cortisol and alpha-amylase. They found no effect on stress hormones, but pupil dilation and subjective judgments showed progress in a 1-h experiment. Perhaps the 2-h TCAD used in the present study was also insufficient to elicit a stress reaction that altered these hormone levels. Further research is required on the short- and long-term relation of LE and LRF on stress hormones, especially when testing for transient fatigue in a laboratory environment.

The d2-R attention and concentration test showed an interaction in processing speed between time (pre- and post-TCAD) and provision (unaided and aided). On a descriptive level, the differences in time implied an increase of processing speed in aided and a decrease in unaided condition. The results include the well-known training effect (Brickenkamp, 1962, 2010) which should be relevant for both conditions, unaided and aided. However, no increase in processing speed was observed in the unaided condition. This can be interpreted by a fatigue-related decrement in performance which overlays the training effect. Contrary, in the aided condition the training improvements remain visible because fatigue and fatigue-related decrements in performance were potentially reduced. This suggests that at the end of the TCAD, HA use reduced LRF compared to unaided testing, allowing greater cognitive resources to be left for the second d2-R test run. Taken together, the data do not confirm hypothesis three (“LRF will lead to a general cognitive performance decrease, also in a visual attention test.”). Hornsby et al. (2016) stated that mental fatigue results in a generalized slowing in processing speed and a decreased ability to maintain attention. Even though the d2-R results did not confirm this relationship, the differences between aided and unaided testing imply that mental fatigue from effortful listening can be reduced through the use of HAs. In future research, the consequences of effortful listening on areas of function outside the auditory domain should be investigated further.

In sum, we conclude that the TCAD is a suitable tool to investigate LRF. Future research with the TCAD will allow more insights into the relationships among LE, LRF, HA provision, and stress hormones.

## Figures and Tables

**Figure A1.**
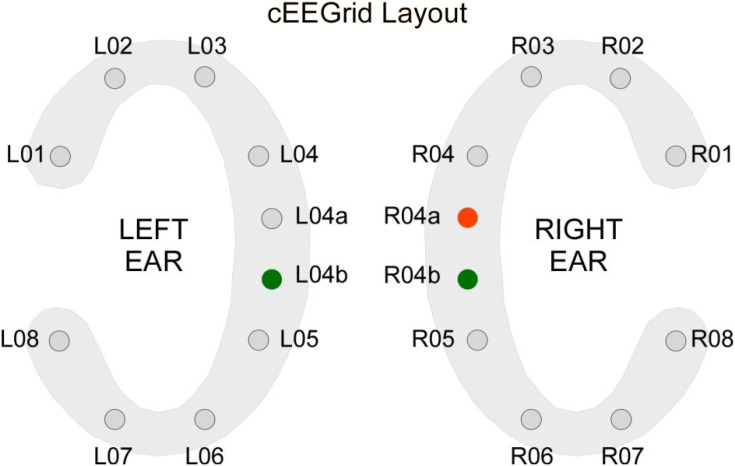
Layout of cEEGrid electrodes that were used in the current experiment. Channel R04a is a hardware reference and channels R04b and L04b are connected within the cEEGrid adapter and constitute the ground.

**Figure A2.**
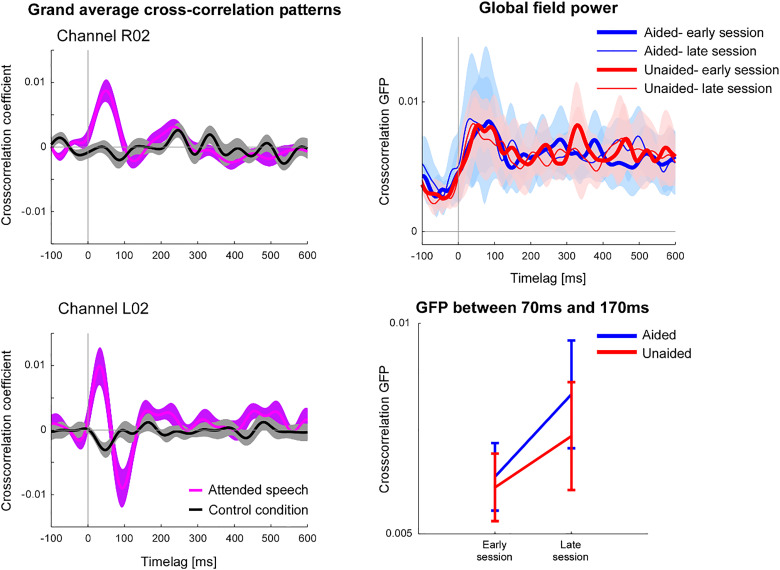
Left: Average cross-correlation patterns on cEEgrid channels R02 and L02 for attended speaker task. Shaded areas represent ± 1 standard error of the mean. The cross-correlation pattern between EEG and corresponding trials of the attended auditory stream is shown in magenta, and the cross-correlation of unmatched EEG and stimulus trials is shown in gray. Upper right: Global field power for aided (blue) and unaided (red) conditions for both daily sessions – first session in thick lines and second session in thin lines. Shaded areas represent ± 1 standard error of the mean. Lower right: ANOVA of condition averages between 70 and 170 ms did not reveal significant condition differences. Error bars represent ± 1 standard error of the mean.

## Appendix

To include EEG as an objective physiological measure, an AAD paradigm was integrated in the TCAD test procedure. The *Attended Speaker* test was designed as an attention task in which subjects had to attend to one auditory stream and ignore another (see the section Attended Speaker—Selective attention comprehension task). As described in the introduction, the AAD relies on neural responses that are cross-correlated to the envelope of a continuous auditory stimulus. The AAD component tends to be higher when a subject is actively attending to an auditory stimulus (compare e.g., to O'Sullivan et al., 2015). Jaeger et al. (2020) found that when LE is reported to be high, the AAD components tend to be less prominent. Additionally, subjects with poor decoding performance showed higher fatigue ratings.

### EEG—Methodology

EEG was used to obtain objective measures of HA benefit on LE and LRF. cEEGrids (Bleichner et al., 2017; Debener et al., 2015) were used instead of the full EEG sensor cap, because the whole cap is cumbersome and may have influenced fatigue measures. cEEGrids are C-shaped electrode arrays placed around the participants’ ears (Figure A1). Each cEEGrid consists of 10 Ag/AgCl electrodes printed on flexible material. Two channels located centrally behind the right ear are designated as reference and ground (R04a and R04b, respectively, Figure A1). For the left ear, the ground electrode is connected to the symmetrical channel (L04b, Figure A1). cEEGrid data was collected at a 500 Hz sampling rate, amplified by the mobile EEG Smarting amplifier (mBrainTrain, Serbia) and sent wirelessly via Bluetooth to the recording computer running a Windows operating system with the Smarting application for EEG data recording. The Smarting application created an LSL stream (Lab Streaming Layer, https://labstreaminglayer.org/) with EEG data sent through an ethernet connection to the TASCAR computer, which recorded the data. Markers that indicated the start of the audio presentation to the participant were sent separately in the LSL marker stream from MATLAB, where they were generated, to the TASCAR computer.

The latency between stimuli (represented by the LSL marker stream) and the recorded EEG was analyzed using a LabStreamer (Neurobehavioural systems, United States). The EEG data were corrected offline for a constant delay by adjusting the timing of all recorded markers. The jitter was on the order of 4 samples (8 ms) for both measurement locations. In subsequent processing steps, EEG data were analyzed using custom MATLAB scripts (R2019a, MathWorks) and EEGLAB toolbox version 13_4_4b (Swartz Center for Computational Neuroscience, SCCN, https://sccn.ucsd.edu/).

Preprocessing: EEG data was first bandpass-filtered between 0.5 and 45 Hz. Infomax-independent component analysis (ICA) was performed, and ICA components were visually assessed. Based on the time course of each component, those related to eyeblinks and electrocardiographic (ECG) artifacts were removed, and the remaining components were back-projected to the data. Artifact subspace reconstruction (ASR), an automatic probability-based artifact attenuation procedure, was additionally performed on the data.

Following the preprocessing steps, EEG data were down-sampled to 250 Hz, filtered with a 15 Hz low-pass FIR filter (Hann window, order 100), and segmented into 20 30-s segments per session. As two sessions of Attended speaker task were performed on each day, the total number of epochs was 120. Epochs that contained amplitudes higher than three standard deviations were rejected. No more than 20% of epochs per participant were rejected.

Analysis: The envelope of presented speech was obtained in a procedure similar to that of Petersen et al. (2017). Original sound streams were Hilbert-transformed and bandpass-filtered between 3 and 25 Hz (third order Butterworth filter). The first derivative of the resulting signal was obtained, followed by half-wave rectification. The result was down-sampled to 250 Hz, the same sampling frequency as the EEG data. Matching the EEG preprocessing, the signal was epoched into 30 s segments.

The focus of the analysis were neural impulse responses, as a lower amplitude of neural impulse response components was shown to be a possible marker for high LE, as well as for LRF. Neural impulse responses (cross-correlation pattern) to presented speech were obtained by cross-correlating corresponding EEG segments and speech-envelope segments at lags from −100 to 600 ms. The control condition was obtained by temporally cross-correlating nonmatching speech-envelope segments and EEG segments. For a more robust comparison between conditions, the global field power (GFP) of cross-correlation patterns was ultimately observed (Holtze et al., 2022).

#### EEG—Results

Statistically meaningful EEG analysis could only be performed for 10 participants, with the remaining 10 eliminated due to noise contamination of traces or the absence of relevant event markers. Figure A2 shows EEG analysis recorded during the attended speaker task. Prominent attention effects on all cEEGrid channels were observed within the period from 70 to 170 ms, reflected by the amplitude difference between cross-correlation of EEG with matching to-be-attended stimulus (test/attended speech condition) and cross-correlation of EEG with nonmatching segments of the stimulus (control condition). The two left panels in Figure 9 show prominent peaks in the neural response at latencies corresponding to the components of the temporal response functions described in the literature (e.g., Mirkovic et al., 2019; Petersen et al., 2017).

Neural impulse responses obtained during listening with and without HA were compared. Additionally, the early and late listening sessions were kept as separate conditions, as LRF may have been higher in the late session. Conditions were compared using GFP as a more robust, channel-independent measure (Figure A2, upper right panel). The mean of GFP values between 70 and 170 ms were compared in a 2 × 2 repeated-measures ANOVA, with factors HA (aided vs. unaided) and time-on-task (early session vs. late session). The time period was chosen as a wide window that encompassed the prominent peaks in the temporal response function, but also limited to periods commonly related to top-down processing. No significant main effects, HA: *F*(1, 9) = 0.682, *p* > .4, time-on-task: *F*(1, 9) = 3.322, *p* > .1, or interactions, *F*(1, 9) = 0.419, *p* > .5, were found (Figure A2, lower right panel).

### EEG—Discussion

EEG was used to gain an objective measure of LRF, and our expectation was to see smaller cross-correlation amplitudes as LRF increased. A general attention effect was observed in the neural traces, reflected by a positive peak around 50 ms and negative trough around 100 ms, confirming participants performed the task correctly. However, we could not find any significant differences between aided and unaided conditions, or time-on-task influences during the day. This may be due to the rather small sample of data that we analyzed, as only a part of the desired data was available due to transmission errors in the recording process. Another possible reason for the lack of an effect could be that mental fatigue may influence one's ability to suppress irrelevant information (Faber et al., 2012; Petersen et al., 2017), rather than enhance the ability to track target information. One limitation of the current study was our inability to analyze the tracking of ignored information, and we would recommend inclusion of a constant audio stream to be ignored in future studies. As meaningful EEG analysis could only be performed for 10 participants, however, the results need to be interpreted cautiously. Nevertheless, our observation of an attention effect in neural impulse responses on all cEEGrid channels supports the AAD approach as a possible marker for the evaluation of attention, LRF, and LE in future studies.
